# A 3D *in vitro* model to explore the inter-conversion between epithelial and mesenchymal states during EMT and its reversion

**DOI:** 10.1038/srep27072

**Published:** 2016-06-03

**Authors:** S. J. Bidarra, P. Oliveira, S. Rocha, D. P. Saraiva, C. Oliveira, C. C. Barrias

**Affiliations:** 1 i3S – Instituto de Investigação e Inovação em Saúde, Universidade do Porto, Rua Alfredo Allen, 208, 4200-135 Porto, Portugal; 2 INEB - Instituto de Engenharia Biomédica, Rua Alfredo Allen, 208, 4200-135 Porto, Portugal; 3 Expression Regulation in Cancer Group, Institute of Molecular Pathology and Immunology of the University of Porto (IPATIMUP), Rua Alfredo Allen, 208, 4200-135 Porto, Portugal; 4 Department of Pathology and Oncology, Faculty of Medicine, University of Porto, Al. Prof. Hernâni Monteiro, 4200-319 Porto, Portugal; 5 Instituto de Ciências Biomédicas Abel Salazar, Universidade do Porto, Rua de Jorge Viterbo Ferreira, 228, 4050-313 Porto, Portugal

## Abstract

Epithelial-to-mesenchymal transitions (EMT) are strongly implicated in cancer dissemination. Intermediate states, arising from inter-conversion between epithelial (E) and mesenchymal (M) states, are characterized by phenotypic heterogeneity combining E and M features and increased plasticity. Hybrid EMT states are highly relevant in metastatic contexts, but have been largely neglected, partially due to the lack of physiologically-relevant 3D platforms to study them. Here we propose a new *in vitro* model, combining mammary E cells with a bioengineered 3D matrix, to explore phenotypic and functional properties of cells in transition between E and M states. Optimized alginate-based 3D matrices provided adequate 3D microenvironments, where normal epithelial morphogenesis was recapitulated, with formation of acini-like structures, similar to those found in native mammary tissue. TGFβ1-driven EMT in 3D could be successfully promoted, generating M-like cells. TGFβ1 removal resulted in phenotypic switching to an intermediate state (RE cells), a hybrid cell population expressing both E and M markers at gene/protein levels. RE cells exhibited increased proliferative/clonogenic activity, as compared to M cells, being able to form large colonies containing cells with front-back polarity, suggesting a more aggressive phenotype. Our 3D model provides a powerful tool to investigate the role of the microenvironment on metastable EMT stages.

Epithelial–to-mesenchymal transition (EMT) is a central process occurring during embryogenesis and wound healing, being also highly implicated in cancer progression^1^,^2^,^3^. During EMT, epithelial (E) cells progressively lose polarity and cell-cell contacts acquiring a mesenchymal (M) phenotype with increased migratory and invasive potential^3^,^4^. EMT confers plasticity to cells, contributing to cell dispersion during development and cancer dissemination^1^,^2^. In epithelial cancers, invading cells display EMT-like features such as a mesenchymal phenotype associated with expression of vimentin (M marker), and loss of epithelial E-cadherin expression, and/or detachment and movement towards the stroma^4^. These cells may undergo the reverse process, mesenchymal-to-epithelial transition (MET), in order to allow growth and colonization at secondary sites, forming metastasis^5^.

Importantly, tumor cells may undergo partial EMT with transitory acquisition of mesenchymal characteristics while retaining epithelial features. These intermediate states, so-called metastable phenotypes, are characterized by phenotypic heterogeneity and cellular plasticity and likely represent the most aggressive clones in a tumor^6^,^7^,^8^. In addition, when cancer cells successfully establish metastasis at secondary sites, they re-acquire E markers while maintaining aggressive tumor features^6^,^7^,^9^. Yet, the study of EMT intermediate stages has been limited by the lack of specific phenotypic markers that hampers identification of these cells *in vivo*^6^,^10^, and by the lack of reliable models to examine inter-conversion between E and M states *in vitro*^8^,^11^,^12^.

To explore the phenotypic and transcriptional switching of cells during EMT, we have previously established an *in vitro* 2D model of transforming growth factor-β1 (TGFβ1)-induced EMT and its reversion^12^,^13^. TGFβ1 supply to the near-normal E cell line EpH4 efficiently generated M-like cells, and its removal resulted in the re-acquisition of an epithelial-like phenotype. The later cellular state, that we named reversed epithelia (RE cells), is characterized by the co-existence of several and heterogeneous cellular populations with regard to the expression of E-cadherin (E marker) or fibronectin (M marker)^13^. In our 2D model, we also demonstrated that RE cells, generated through MET, together with heterogeneity display increased mamosphere formation efficiency and *in vivo* tumourigenesis ability^13^. RE cells, unlike E and M, possibly reproduce tumor heterogeneity often described in primary and metastatic clinical samples^8^,^11^. Still, traditional 2D models are reductionist, since they fail to recapitulate key architectural features of native tissues, namely in what concerns the impact of the extracellular matrix mechanical and biochemical properties^14^. The paradigm shift from 2D to 3D culture is underway and progressing rapidly, being currently recognized that adding the 3^rd^ dimension to a cell’s environment creates significant differences in cellular characteristics and function^15^. M Bissel’s team elegantly demonstrated the relevance of using 3D systems to investigate cancer mechanisms, by creating a prototypical model of the mammary gland acinus, where TGFβ1-induced EMT occurred^16^. 3D models where cells are completely surrounded by a supportive 3D matrix, i.e. hydrogel-based entrapment systems, are the most relevant systems for modulating cell-matrix interactions^17^,^18^,^19^. Extracellular matrix (ECM)-derived protein gels such as collagen or Matrigel^TM^ are commonly used, but generally present poorly tunable biochemical/biomechanical properties, high batch-to-batch variability and intrinsic bioactivity, which makes it very difficult to compare results between different Laboratories, and even between different experiments^18^,^20^. More recently, biomaterial-based platforms, traditionally associated with tissue engineering approaches, have been translated into cancer research creating improved models to study tumor biology, where matrix bioactivity and mechanical properties can be more easily controlled^18^,^19^,^21^,^22^.

In this work, our 2D model evolved towards a new 3D *in vitro* model, by combining the inducible epithelial cell line (EpH4)^12^,^13^ and a bioengineered ECM-like matrix with independently tunable properties, to explore the inter-conversion between E and M states during EMT and its reversion (MET). The selected 3D matrix, composed of an optimized soft alginate hydrogel functionalized with cell adhesive RGD peptides^23^,^24^, supported epithelial morphogenesis, promoting the formation of acinar-like structures similar to those present in mammary tissue, and allowed TGFβ1-induced generation of cells with mesenchymal-like and intermediate phenotypes, providing a useful tool to unravel cellular alterations associated with EMT/MET.

## Results

### 3D culture in soft RGD-alginate matrices preserves the epithelial phenotype of normal mammary EpH4 cells and promotes epithelial morphogenesis

**Figure 1:**
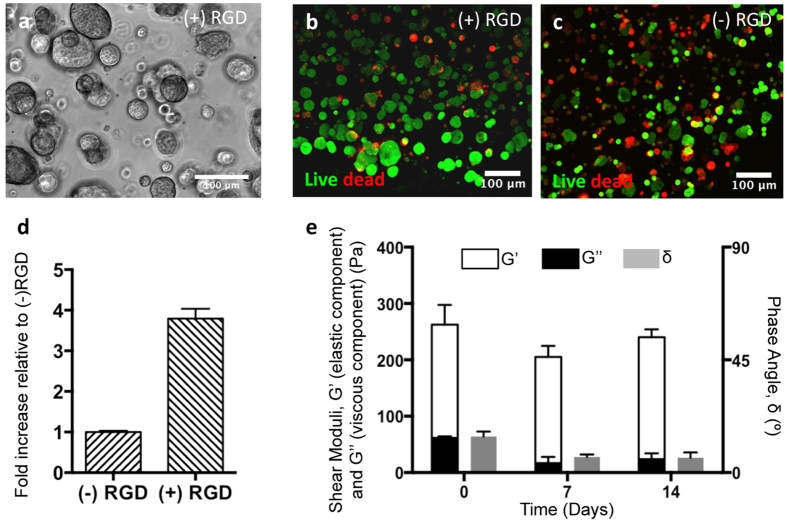
(**a**) Bright field image of EpH4 cells culture in 3D alginate matrices with 200 μM of RGD (+) during 14 days. Viability of Eph4 within 3D alginate matrices (**b**) with RGD (+) and (**c**) without RGD (−) during 14 days. Live cells are stained by calcein AM (green) and dead cells by ethidium homodimer-1 (red). Scale bars: 100 μm. (**d**) Metabolic activity of EpH4 cultured in 3D alginate matrices with (+) and without (−) RGD peptides after 7 days in culture. Data normalized for metabolic activity values obtained for cells in 3D alginate matrices without RGD. (**e**) Viscoelastic properties (elastic, G’ and viscous, G” components of the shear moduli, and phase angle, δ) of 1 wt.% RGD-alginate with EpH4 cells during 14 days of culture. Data are presented as mean ± standard deviation (n = 3).

Soft alginate hydrogels functionalized with cell-adhesion RGD peptides were used in this study to simulate the 3D microenvironment of normal mammary tissue. To determine the best culture conditions for EpH4 cells, these were cultured along 14 days in alginate 3D matrices (i) with and without RGD, (ii) with different stiffness (G’ ≈ 200 Pa with 1 wt.% alginate, G’ ≈ 3000 Pa with 2 wt.% alginate) and (iii) at different cell densities (1 × 10^6^, 5 × 10^6^ and 10 × 10^6^ cells/mL). From these preliminary assays (some data not shown), softer alginate hydrogels with 200 μM RGD and a cell density of 5 × 10^6^ cells/mL showed to be the best conditions for culturing EpH4 cells in 3D, namely by enabling the formation of larger multicellular spheroids with higher cell viability (Fig. 1a–c). The presence of tethered cell-adhesion RGD ligands in the matrix, at a density of 200 μM, similar to that found in common ECM-derived biological matrices^25^, was essential, leading also to higher cell metabolic activity (Fig. 1d). The stiffness of the softer hydrogels (G’ ≈ 200 Pa) was comparable to that of normal mammary tissue^26^,^27^,^28^, and remained essentially unchanged along the culture period (Fig. 1e), as demonstrated by rheological analysis of cell-laden 3D matrices.

**Figure 2:**
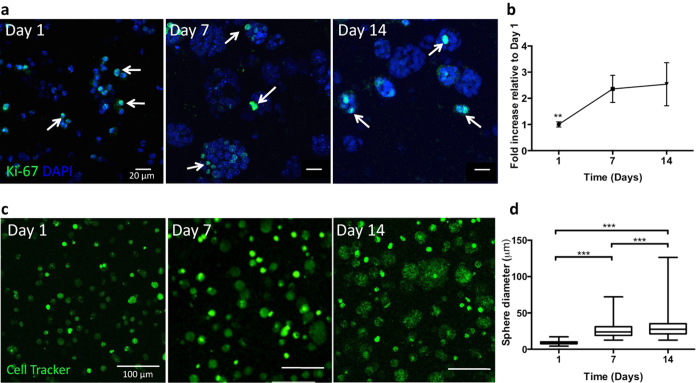
Behavior of normal mammary EpH4 epithelial cells within an artificial 3D RGD-alginate matrix. (**a**) Proliferating epithelial cells (Ki-67 positive cells, arrows) were detected within the matrix at all time points (scale bars: 20 μm). (**b**) The metabolic activity profile showed a significantly increase after 1 week of culture (n = 3) (**c**) Eph4 cells (labeled with CellTracker^TM^ green) formed spheroids that increased in size and number along 14 days of culture (scale bar: 100 μm). (**d**) After 14 days of culture, spheroids reached an average diameter of 20 μm (n = 1876 spheroids). Data is presented as mean ± standard deviation. Statistical significance, **p < 0.01, ***p < 0.001.

Immunodetection of proliferative cells (Ki67 proliferation marker) showed that EpH4, which were initially distributed as single cells within the 3D matrices, were able to proliferate, generating spheroids. At day 1, Ki-67 staining depicted proliferation in individual cells, but at days 7 and 14 proliferative cells were essentially restricted to spheroids (Fig. 2a). The analysis of mitochondrial metabolic activity (Fig. 2b) showed a significant increase along the first week of culture, suggesting that cells were actively proliferating, while from day 7 to day 14 no significant differences were observed. As time progressed, spheroids size increased reaching an average diameter of around 30 μm by day 14 (Fig. 2c,d).

**Figure 3:**
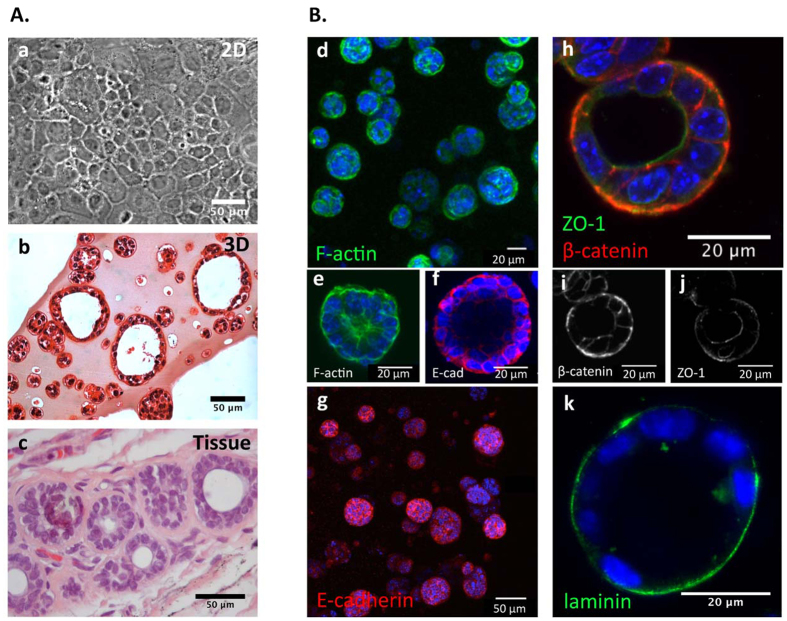
(**A**) Bridging the gap between 2D and tissue. (a) Phase contrast microscopy image of EpH4 cells growing in polystyrene forming a 2D monolayer. Hematoxylin and eosin stained formalin-fixed and paraffin-embedded sections of (b) EpH4-laden 3D alginate matrices after 14 days in culture; and (c) normal breast tissue. Alginate-based 3D *in vitro* model recapitulates the structural architecture (acinar-like structures) of normal breast tissue, supporting epithelial morphogenesis. (**B**) CLSM images of 3D-cultured epithelial cells at day 14 (d and g) composed of 22 Z-stacks projected onto a single plane (representing a total thickness of 105 μm) show spheroids formation and images of 1 Z-stack from the central part of the spheroid revealed lumenization (e,f,h,I,j). (d,e) Eph4 stained for F-actin (green) and nuclei (blue). (f,g) Expression of the classical epithelial marker E-cadherin (red) and nuclei (blue). (h) 3D culture EpH4 show sphere lumenization and polarization, staining for basolateral marker β-catenin (red, (i)) and for apical marker ZO-1 (green, (j)). (k) Immunostaining for laminin (green) show that entrapped cells were able to secrete laminin. Scale bars (a,b,c,g) 50 μm and (d,e,f,h,i,j,k) 20 μm.

Normal murine mammary epithelial EpH4 cells that typically assume a polygonal or cuboidal shape in 2D monolayer culture, with forced, non-physiological cell polarization (Fig. 3a), assembled into large spheroids when cultured in soft RGD-alginate 3D matrices (Fig. 3b). This 3D arrangement mimics the typical cell/matrix organization found in normal mammary tissue (Fig. 3c), being histologically identical. Structures formed by EpH4 cells in 3D were classified according to five categories, as proposed in^29^, namely: (I) immature with few cells, (II) spherical with a filled lumen, (III) spherical with a hollow lumen, (IV) non-spherical but organized, or (V) non-spherical and disorganized. After 12 days of culture, only structure classes I-III were observed. Around 20% of those structures already presented a cleared lumen (Supplementary Fig. S1), resembling mammary acini, the basic anatomical units of the mammary gland.

F-actin and E-cadherin staining of EpH4-laden hydrogels, also revealed the formation of uniform spheroids after 10–14 days in 3D culture (Fig. 3d–g). Lumenized structures (Fig. 3e,f) showed peripheral nuclear alignment and apical-basal polarity maintained by the precise arrangement of actin filaments (Fig. 3e)^30^, namely at interior luminal surface and at cell-cell junctions, and stained positively for functional E-cadherin (Fig. 3f), a prototypical epithelial marker, which was localized at the cell membrane, stabilizing cell-cell contacts within spheroids. Furthermore, lumenized spheroids showed segregation of polarity markers (Fig. 3h–j): basolateral β-catenin was nearly absent on the apical side, whereas zonula occludens-1 (ZO-1) was notably present, although with some persistence at the basal side. Importantly, cells were able to assemble a laminin-rich layer around spheroids, at the basal edge (Fig. 3k).

**Figure 4:**
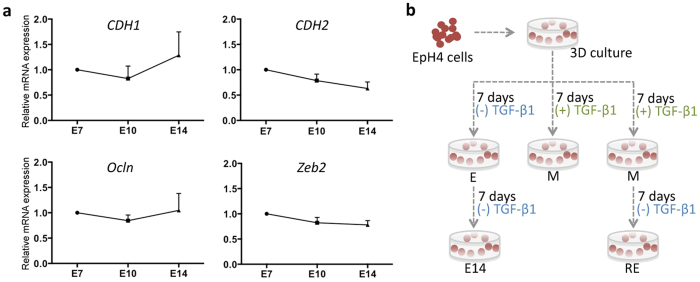
(**a**) qRT-PCR quantification of relative mRNA expression of *CDH1* and *Ocln* (E markers), *CDH2* (M marker) and *Zeb2* (EMT inducer). Expression of the different markers was not significantly altered during the 14 days of culture. Data normalized for E cells and presented as mean ± standard deviation (n = 4 biological replicas). (**b**) Schematic representation of TGFβ1-driven EMT and its reversion in a 3D *in vitro* model. EpH4 were immobilized within 1 wt.% alginate matrix biofunctionalized with 200 μM RGD. To study epithelial morphogenesis, cells were kept in standard culture medium during 14 days (E cells). For EMT induction, medium was supplemented with TGFβ1 during 7 days (M cells). To revert the attained phenotype, TGFβ1 was removed and cells were maintained in culture for another week (RE cells).

By qRT-PCR, we assessed the mRNA expression of epithelial markers, *CDH1* (encoding E-cadherin) and *Ocln* (encoding Occludin); mesenchymal marker *CDH2* (encoding N-cadherin); and transcription factor *Zeb2*, a well-known EMT inducer (Fig. 4a). None of the assessed markers were significantly altered across time, suggesting that 3D culture within RGD-alginate hydrogels preserves EpH4 cells epithelial phenotype and supports normal epithelial morphogenesis.

### TGFβ1 induces EMT in normal mammary epithelial EpH4 cells cultured under 3D conditions and its removal generates cells with intermediate phenotype

**Figure 5:**
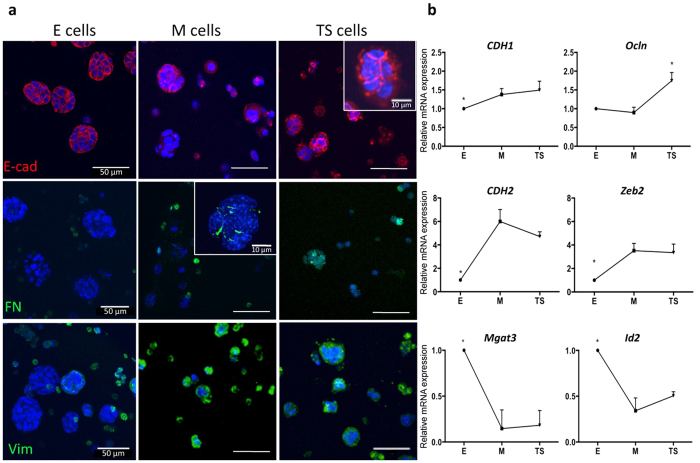
Expression of E and M markers during 3D EMT and its reversion, at mRNA and protein levels. (**a**) At protein level, E cells display the classical E-cadherin expression at cell membrane; M cells show decreased E-cadherin expression and delocalization into the cytoplasm; and RE cells display recovery of E-cadherin expression at the cell membrane. E cells did not express fibronectin (green), while M cells not only expressed intracellular fibronectin but also assembled pericellular fibronectin within small cellular aggregates (inset). RE cells showed decreased fibronectin expression, as compared to M cells, but higher expression that E cells. The higher levels of vimentin expression (green) were detected in M cells, with RE cells presenting intermediate expression levels as compared with E and M cells. Cell nuclei, blue. Scale bars: 50 μm (inset images: 10 μm). (**b**) At mRNA level, *CDH1* (E marker) expression was increased in M and RE cells, as compared with E cells; *Ocln* (E marker) slightly decreased in M cells and was recovered in RE cells; *CDH2* (M marker) was significantly increased in M cells and then slightly decreased in RE cells; *Zeb2* (EMT inducer) was significantly increased in M cells as compared with E cells, supporting EMT occurrence; *Mgat3*, an epithelial-associated marker, was significantly decreased in M cells and slightly increased in RE cells (in comparison with M cells); and *Id2* (negative regulator of TGFβ-induced EMT), was significantly decreased in M cells and then increased in RE cells. Data was normalized for E cells and presented as mean ± standard deviation (n = 4 biological replicas, from 4 independent experiments). Statistical significance, *p < 0.05.

Having established that 3D culture within RGD-alginate hydrogels did not promote EMT *per se*; we next induced EMT by exposure to soluble TGFβ1 (Fig. 4b)^12^,^13^. Media supplementation with 16 ng/mL TGFβ1 (Fig. 5a) generated M-like cells from E cells, after 7 days in culture. This concentration had to be optimized relatively to our previous 2D model, where a TGFβ1 concentration of 8 ng/mL had been used. As a control, we used EpH4 cells cultured for 7 days in standard culture media (E cells). As observed by immunofluorescence (Fig. 5a), M cells presented decreased E-cadherin (E-cad) expression (as compared to E cells), and delocalization from the cell membrane to the cytoplasm, suggesting impaired functionality as cell-cell adhesion molecule. M cells also expressed typical mesenchymal markers, namely fibronectin (FN) and vimentin (Vim). Importantly, M cells not only expressed intracellular fibronectin but also assembled pericellular fibronectin within multicellular aggregates (Fig. 5a).

Removal of TGFβ1 from the culture medium for an additional week, led to partial phenotypic reversion from a mesenchymal-like to an epithelial-like state (RE cells), in which E-cad expression at cell membrane was recovered, while expression of M markers (FN and Vim) was still present (Fig. 5a). To better examine whether the observed phenotypic alterations were due to EMT and its reversion, we next analyzed mRNA expression by qRT-PCR (Fig. 5b) of several relevant markers. The mRNA expression of *CDH1* (E marker) remained unchanged across the experiment, while *Ocln* expression (E marker) was only significantly increased in RE cells (*p* = 0.05). Expression of the mesenchymal marker *CDH2* and the EMT inducer *Zeb2*, was significantly increased upon EMT induction (M cells) (*p* = 0.0286 for *CDH2*, *p* = 0.0286 for *Zeb2*), and remained elevated in RE cells. Expression of *Mgat3*, an epithelial-associated marker^12^,^31^, was significantly decreased in M cells (*p* = 0.0286) and remained at low levels in RE cells (*ca.* 2-fold). Finally, mRNA expression of *Id2* (inhibitor of differentiation 2), which is considered as a key negative regulator of TGFβ1–induced EMT in epithelial cells^32^, was significantly decreased in M cells, as compared to E cells (*p* = 0.0286), slightly recovering in RE cells. Overall, expression of EMT markers in E, M and RE cells, at protein and gene levels, point to the occurrence of TGFβ1–induced EMT and partial reversion to an epithelial-like phenotype in 3D, as observed in our 2D model^12^,^13^.

### Inter-conversion between epithelial and mesenchymal phenotypes during TGFβ1–induced EMT and its reversion in 3D

**Figure 6:**
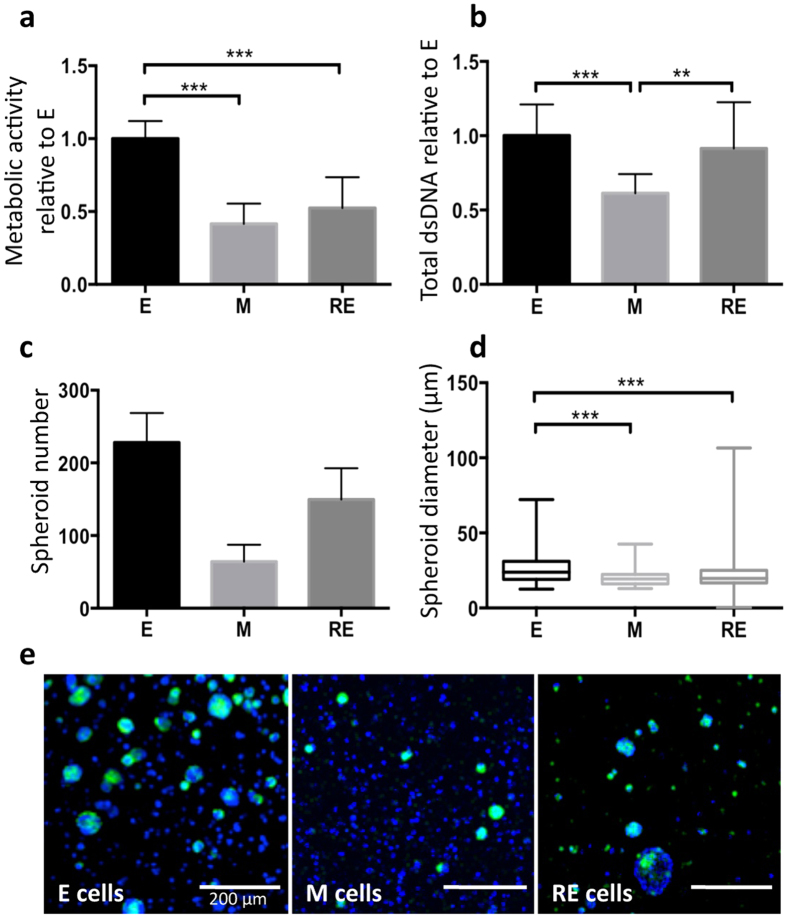
Morphological and functional features of E, M and RE cells in 3D. (**a**) The metabolic activity and (**b**) total dsDNA profiles showed a significantly decrease in M cells, followed by a significantly increase in RE cells. Results were normalized for E cells (n = 3 biological replicates from 3 independent experiments). (**c**) Quantification of spheroid number and (**d**) spheroid diameter (3 replicas per condition and a total of 1316 spheroids). (**e**) Representative CLSM images of Eph4 spheroids during EMT and its reversion. Cell nuclei: blue, F-actin: green. Scale bars: 200 μm. All data were presented as mean ± standard deviation. Statistical significance, *p < 0.05, **p < 0.01, ***p < 0.001.

To better understand the inter-conversion between epithelial and mesenchymal states in our 3D model, we further characterized the behavior of E, M and RE cells at different levels. E cells presented the highest levels of mitochondrial metabolic activity (Fig. 6a) and total dsDNA content (Fig. 6b), indicating a higher proliferative activity. M cells showed a significant decrease (*ca.* 2-fold) of both parameters (*p* = 0.002 and *p* < 0.0001, respectively), supporting growth-arrest promoted by TGFβ1. Finally, RE cells presented intermediate values, implying that EMT reversion allow cells to partially recover their proliferative activity (*p* = 0.0038 for total dsDNA content). Analysis of multicellular spheroid formation (Fig. 6c–e), largely related with clonal cell growth, provided further insights into E, M and RE cells proliferation patterns. E cells formed higher numbers of spheroids, with higher average diameter, as compared to M and RE cells. In contrast, M cells formed significantly less and smaller spheroids, while RE cells generated an intermediate number of spheroids, but with a large diameter variation. Noteworthy, the largest spheroids observed across the 3D model occurred in RE cells, with diameters reaching up to 100 μm.

### E, M and RE cells express different levels of MMPs activity and display different invasive behaviors

**Figure 7:**
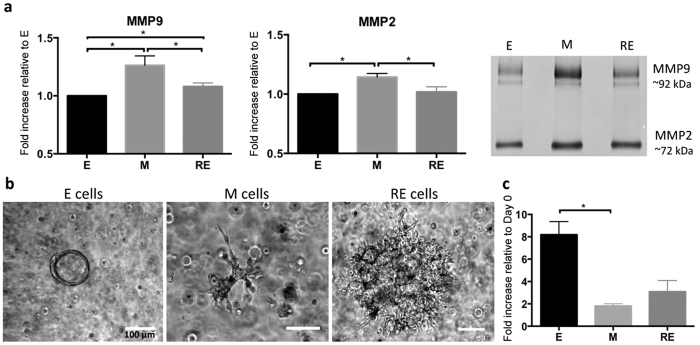
E, M and RE cells express different levels of MMPs activity and display different invasive behavior. (**a**) Activity of MMP2 and MMP9 secreted by 3D-cultured E, M and RE cells analyzed by gelatin zymography. Both MMPs were significantly increased in M cells; RE cells showed a significantly decrease in both MMPs in comparison with M cells, albeit MMP9 secretion was significantly higher in RE than in E cells. Data was normalized to E cells and presented as mean ± standard deviation (n = 3 biological replicates from 3 independent experiments). Statistical significance, *p ≤ 0.05. (**b**) Bright-field images of E, M and RE cells cultured for 7 days in Matrigel^TM^ after recovery from RGD-alginate 3D matrices. E cells formed organized spheroids with lumen, while M and RE cells formed star-like structures (associated with an invasive phenotype), which were larger in RE cells. Scale bars: 100 μm. (**c**) Fold increase (in relation to day 0) in E, M and RE cells metabolic activity after 1 week of culture in Matrigel^TM^: E cells showed the highest increase in metabolic activity, followed by RE cells and then M cells. Data normalized for cells at day 0 and presented as mean ± standard deviation (n = 3 replicas).

Cells undergoing EMT are known to acquire a migratory phenotype and increased invasion capacity, features also seen in tumor cells that invade and metastasize. Expression of matrix metalloproteinases (MMP) activity is known to play a key role in these processes, namely in ECM degradation^33^,^34^. Here, we quantified the secretion of MMP2 and MMP9 (Fig. 7a) and our results show that both were significantly increased in M cells (as compared to E cells, *p* = 0.05). Secretion of both MMPs was significantly decreased in RE cells, when compared to M cells (*p* = 0.05).

To evaluate their invasion potential, E, M and RE cells were recovered from RGD-alginate 3D matrices and entrapped in Matrigel^TM^ for 7 days. Each of the three states displayed unique morphological features. Organized spheroids with lumen, suggestive of a non-invasive phenotype (Fig. 7b), were only detected in E cells, which also presented the highest metabolic activity (Fig. 7c, *p* = 0.05). In both M and RE cell cultures, multicellular aggregates with a star-like appearance and containing cells with spindle-like morphology were detected (Fig. 7b), which have been associated with invasive phenotypes^35^,^36^. The size of such structures was much larger in RE cells (as compared to M cells), which concomitantly presented higher metabolic activity (Fig. 7c), suggesting higher proliferative activity. Altogether, these results show that RE cells generated in our 3D model, present an intermediate phenotype, combining mesenchymal and epithelial features.

## Discussion

In this report, we describe a new 3D *in vitro* model, combining an inducible epithelial cell line (EpH4)^12^,^37^ with a bioengineered ECM-like matrix, to explore transitions between epithelial and mesenchymal phenotypes. Current interest in these processes stems from their well-documented involvement in cancer dissemination^5^,^38^. Different reports experimentally support the idea that EMT reversion is key for successful colonization and metastasis of different types of cancers^39^,^40^. Yet, while EMT has been largely studied by examining “pure” epithelial or mesenchymal states, transient (metastable) phenotypes, still remain poorly understood, especially because there are quite difficult to capture *in vivo*^6^,^10^. By creating greater similarity with *in vivo* scenarios, as compared to monolayer 2D models, 3D models of EMT and its reversion are likely to generate instrumental tools to mechanistically understand these intermediate phenotypes. These models may allow the identification of novel players with relevance in cancer progression and therapy.

In this study, an alginate-based hydrogel matrix was used to recreate a suitable 3D microenvironment for culturing EpH4 cells, capable of mimicking the stromal component of mammary tissue. Alginate is a natural polymer able to form hydrogel matrices that are essentially bio-inert, in the sense that they do not elicit specific cell-matrix interactions. However, alginate chains can be covalently modified with bioactive moieties, namely with peptides containing the amino acid sequence arginine-glycine-aspartic acid (Arg-Gly-Asp, RGD) to promote integrin-mediated cell adhesion and cell-matrix crosstalk, as already demonstrated for other types of hydrogels^29^. Moreover, alginate concentration and molecular weight can be easily altered to modulate the viscoelastic properties and degradation rate of the resulting hydrogels^41^. Here, to provide physiologically-relevant biochemical and biomechanical cues, we fine-tuned the properties of alginate hydrogels to attain a density of cell-adhesion ligands (200 μM)^25^ similar to that present in common ECM-derived biological matrices and viscoelastic properties (G’ ≈ 200 Pa) comparable to that of normal breast tissue and mammary gland^26^,^27^,^28^. We also monitored the evolution of hydrogel stiffness along time, to guarantee that it would remain unchanged throughout the culture period, as several studies have demonstrated that soft matrices are protective against EMT, whereas stiffer matrices may promote EMT inducing malignant phenotypes in normal mammary epithelial cells^42^,^43^.

In optimized RGD-alginate 3D matrices, single EpH4 cells were able to proliferate, forming spheroids by clonal-growth as suggested by the patterning of Ki67 staining. This was further demonstrated by pre-labeling EpH4 cells with fluorescent dyes of different colors, and by observing the formation of single-color spheroids after same days in culture (Fig. S2). We also observed, to a lesser extent, multi-color spheroids likely derived from early aggregation of labeled single cells in close proximity, or some kind of inter-cellular dye exchange resulting in dual fluorescence labeling^44^ (Fig. S2). Along the time of culture, these multicellular structures maturated into spheroids with a hollow central lumen, which recapitulate mammary acini strcuturee. They presented typical features, such as growth arrest, apico-basal organization with segregation of polarization markers and deposition of an endogenous laminin-rich matrix layer at the basal edge, mimicking the native basal lamina. Noteworthy, correct cell polarization is a clear requirement of epithelial cells models, since polarity influences intracellular signal trafficking, gene expression and phenotype^45^. Altogether, these results validate our model in its capacity to efficiently recapitulate normal epithelial morphogenesis, as already reported using both natural and synthetic, yet more complex, 3D matrices^16^,^22^,^46^. The maintenance of mRNA expression levels of typical E markers (*CDH1*, *Ocln*), with no up-regulation in expression of M markers (*CDH2*) and EMT inducers (*Zeb2*), demonstrated that 3D culture within soft RGD-alginate matrices does not induce EMT *per se*^47^,^48^. Overall, by promoting *ex vivo* organotypic cellular organization under controlled experimental conditions, our *model* provides a relevant platform for mimicking the microenvironment of epithelial tumors, since epithelial cells grown as monolayers on plastic surfaces bear little to no resemblance to their *in vivo* counterparts^45^.

After establishing a reliable 3D platform for normal epithelial morphogenesis, we then demonstrated that EMT could be successfully promoted upon TGFβ1 treatment, giving rise to M cells, as previously described in our previous 2D model^12^,^13^. Key EMT-associated phenotypic alterations were clearly identified in our 3D model, such as loss of cell-cell adhesions (loss of functional E-cadherin) and expression/deposition of the ECM protein FN and the cytoskeleton protein Vim, indicating a switch towards a mesenchymal-like phenotype. EMT response downstream of TGFβ1 signaling is induced by transcriptional reprogramming, promoting activation of genes encoding mesenchymal proteins such as N-cadherin, which renders cells more motile and invasive, as well as transcriptional factors such as *Zeb2*, both of which were up-regulated in our 3D model^49^,^50^. We also observed a significantly down-regulation in mRNA expression of *Mgat3* and *Id2* in M cells. *Mgat3* encodes for N-acetylglucosaminyltransferase III (GnT-III), which modulates the glycosylation state of E-cadherin in epithelial *adherens-junctions*, leading to E-cadherin internalization and disruption of cell-cell contacts^12^,^51^. We previously showed that *Mgat3* expression was dramatically decreased during EMT in 2D, and later recovered when cells partially returned to an epithelial-like phenotype, and identified *Mgat3* glycogene and GnT-III mediated glycosylation (specifically on E-cad) as a novel and major component of the EMT mechanism signature. On the other hand, *Id2* is a member of the helix-loop-helix (HLH) protein family that has been described as an EMT antagonist, maintaining epithelial differentiation. Strong suppression of *Id2* expression has been observed during EMT in different epithelial models, and forcing *Id2* expression in mesenchymal cells has been shown to partially rescue an epithelial phenotype^32^,^52^,^53^,^54^. Therefore, the significant decrease in *Mgat3* and *Id2* expression, as observed here, further points to the occurrence of EMT in our 3D model.

Removal of TGFβ1 clearly resulted in (partial) EMT reversion, generating what we called RE cells, a hybrid cell population endowed with mixed epithelial and mesenchymal characteristics. Phenotypically, these cells recovered functional E-cad protein expression at cell membrane, however retaining concomitant expression of characteristic mesenchymal proteins (FN and Vim). Importantly, RE cells also displayed an intermediate mRNA expression profile for the genes described above, in particular RE cells displayed a significant up-regulation of the epithelial marker *Ocln*, while retaining high levels of expression of mesenchymal markers, such as *CDH2* and *Zeb2*. These observations support the intermediate nature of this cellular state^12^,^13^.

The phenotypic alterations occurring during EMT and its reversion in 3D reflect different functional properties of E, M and RE cellular states. It has being described that TGFβ1 is not only responsible for EMT induction but also is capable to arrest the mammary epithelial cell cycle^55^. Growth-arrest is currently considered as one of the hallmarks of EMT, allowing cells to (de)differentiate, acquire a mesenchymal-like phenotype and become motile and invasive^56^,^57^. Also, it has been reported that EMT-inducing transcriptional factors can directly inhibit cell proliferation^3^. In our 3D model, proliferation was in fact decreased in M cells, which also presented higher levels of MMP2 and MMP9 activity, known to play a key role in initiating localized matrix degradation at basement membrane, during epithelial cancer invasion^34^. On the other hand, it has also been proposed that EMT reversion is a driving force of metastasis, being necessary for the re-acquisition of proliferative activity and colonization capacity^56^. The RE cells population generated with our 3D model recovered some proliferative activity, as compared to M cells, showing ability to form very large multicellular spheroids, and also displayed slightly higher levels of MMPs activity than E cells. Moreover, E, M and RE cells recovered from 3D culture also exhibited strikingly different behaviors when entrapped in Matrigel. While E cells were able to form structures resembling organotypic mammary acini with apical-basal polarity, M and RE cells formed more disorganized cell spheroids, containing cells with front-back polarity, a feature that has been associated to malignant phenotypes^35^,^36^. However, only RE cells showed ability to form very large spheroids, forming complex structures^35^. This further supports that RE cells obtained in our 3D model present features of intermediate phenotypes, likely containing heterogeneous cell subpopulations with high clonogenic capacity and ability to form large colonies, while maintaining invasive capacity, hallmarks of a more aggressive phenotype.

In summary, in our 3D model we were able to generate a hybrid phenotype of RE cells that clearly shares features from both E and M cells, which has been correlated with a more aggressive behavior. Although capturing EMT transient stages *in vivo* has been challenging, namely due to current lack of reliable markers and readouts, a few recent studies suggest that cells in hybrid E/M or partial EMT state are effectively most likely to give rise to more aggressive tumor clones, as opposed to cells in pure epithelial (E) or pure mesenchymal (M) states. The 3D-RE cells that we herein generated share features with intermediate or hybrid EMT states observed *in vivo*, namely: 1) epithelial morphology reminiscent of E cells and invasive features similar to M cells; 2) concomitant expression of epithelial and mesenchymal markers (high *CDH1* and *Ocl* and high *CDH2* and *Zeb2*), and; 3) sustained expression of the E-cadherin repressor *Zeb2*. These observations support an association with more aggressive phenotypes as described by Strauss *et al.*, who showed that some cells in hybrid E/M phenotype in primary ovarian cultures and tumors *in situ* can express heterogeneous lineage markers, and drive tumor growth *in vivo* by giving rise to another E/M subset, as well as completely differentiated epithelial cells^58^. In another study, Ruscetti *et al.* isolated hybrid E/M cells *in vivo* in a prostate cancer mouse model and demonstrated their comparable or even higher tumor-initiating potential as compared to full mesenchymal cells^59^. Our system thus stands as a valuable 3D *in vitro* model to generate and investigate cells with metastable phenotypes, which are highly relevant.

The proposed system is highly versatile, as it allows independent tuning of several matrix features, such as adhesiveness, stiffness and degradability, as previously described by our group^23^,^24^, which can be used to modulate cell-cell and cell-matrix interactions in a strictly controlled way. This way, in future studies, it may be easily optimized for culturing other relevant cell lines, namely normal and/or cancer human cells. The proposed 3D model is thus expected to provide a valuable tool for mechanistically defining molecular characteristics of invasive cells and their surrounding matrix. This will pave the way for the development of new anti-cancer therapies targeting, for example, the reversion of metastable phenotypes by stabilizing the non-invasive epithelial phenotype^60^.

## Conclusions

A new 3D *in vitro* platform for generating and tracking intermediate stages of cancer-associated EMT was developed. Soft RGD-alginate matrices supported normal epithelial morphogenesis, while allowing TGFβ1–induced EMT and its reversion, which generated cells with epithelial-like, mesenchymal-like and, most importantly, intermediate phenotypes. The proposed bioengineered matrices provide simple, yet biologically meaningful, cellular microenvironments, where cell-matrix interactions can be precisely modulated in a systematic way. Their use as a 3D *in vitro* model for studying metastable EMT stages is expected to improve current knowledge towards identification of putative targets for more effective anti-cancer therapies.

## Materials and Methods

### Synthesis and characterization of RGD-alginate

Ultrapure sodium alginate (PRONOVA UP LVG, Novamatrix, FMC Biopolymers) was covalently grafted with the cell-adhesion peptide (glycine)4-arginine-glycine-aspartic acid-serine-proline (hereafter abbreviated as RGD) using aqueous carbodiimide chemistry as previously described^61^. Briefly, alginate solutions (1 wt.%) in MES buffer (0.1 M MES, 0.3 M NaCl, pH 6.5) were prepared and stirred overnight (ON) at room temperature (RT). N-Hydroxy-sulfosuccinimide (Sulfo-NHS, Pierce) and 1-ethyl-(dimethylaminopropyl)-carbodiimide (EDC, Sigma, 27.4 mg per g of alginate) were sequentially added at a molar ratio of 1:2, followed by 100 mg of RGD peptide (Genscript) per g of alginate. After stirring for 20 h at RT, the reaction was quenched with hydroxylamine (Sigma) and the solution was dialyzed against deionized water for 3 days (MWCO 3500). After purification with charcoal, RGD-alginate was lyophilized and stored at −20 °C until further use. The reaction yield was calculated using the BCA Protein Assay (Pierce), as previously described in^62^.

### Preparation and characterization of RGD-alginate hydrogel matrices

*In situ* forming alginate hydrogel matrices were prepared by internal gelation as described previously^23^,^24^,^62^. Hydrogel precursor solution was prepared at 1 wt.% sodium alginate in 0.9 wt.% NaCl, with 200 μM RGD, a density comparable to that present in commonly used ECM-derived biological matrices^25^. The solution was sterile-filtered (0.22 μm) and mixed with an aqueous suspension of sterile CaCO_3_ (Fluka) at a CaCO_3_/COOH molar ratio of 1.6^62^. Then, a fresh sterile solution of glucone delta-lactone (GDL, Sigma) was added to trigger gelation. The CaCO_3_/GDL molar ratio was set at 0.125, and the gelation time was 45 min.

Rheological measurements were carried out using a Kinexus Pro rheometer (Malvern). Hydrogel discs were analyzed at day 0 (after swelling to equilibrium in culture media for 1 h) with and without cells under standard culture conditions. To guarantee the dimensional homogeneity of the samples, 8 mm cylindrical discs were cast, and then 4 mm cylinders were punched from the original ones immediately before analysis. All samples were assayed using a plate-and-plate geometry (4 mm diameter, sandblasted surfaces) and were compressed to 20% of their original thickness to avoid slippage. A solvent trap was used to minimize sample drying. All measurements were performed at 37 °C (Peltier system). Stress sweeps (0.1 Hz) were first performed to determine the LVR. Frequency sweeps (0.01–2 Hz) were then performed within the LVR. The values of the shear moduli (G’ and G”) and phase angle, were obtained at a frequency of 0.1 Hz. Samples were analyzed in triplicate.

### 3D culture of EpH4 cells and TGFβ1-driven EMT induction/reversion

EpH4 cell line was kindly provided by Dr. Angela Burleigh and Dr. Calvin Roskelley: from British Columbia Cancer Agency, Vancouver, Canada. EpH4 authentication was performed at the Ipatimup’s Cell Lines Bank, using STR amplification (Promega-Powerplex16, Identifiler). For cell entrapment, EpH4 cells^37^ (5 × 10^6^ cells/mL) were combined with RGD-alginate solution and crosslinking agents and the mixture was pipetted (20 μL) onto Teflon plates separated by 750 μm-height spacers. For spheroid quantification discs with 8 μL and 250 μm height were made. After gelation, 3D matrices were transferred to pHEMA-treated 24-well culture plates. Thereafter, fresh medium was added and renewed after 1 h. 3D culture of EpH4 (classified as E for epithelial) were maintained in DMEM/F-12 with glutamax (Gibco) supplemented with 5% v/v FBS (Biowest), 1% v/v penicilin/streptomycin (Gibco) and 5 μg/mL insulin solution human (Sigma). To induce EMT in 3D cultured EpH4 cells (Fig. 4b), culture medium was supplemented with 16 ng/mL of TGFβ1 during 7 days to generate mesenchymal-like cells (classified as M for mesenchymal). The culture medium was changed every other day with fresh TGFβ1. To revert the EMT process (Fig. 4b), 3D culture of M cells was maintained in non-supplemented culture medium for another 7 days to generate cells with intermediate phenotype (classified as RE for reversed epithelia). To guarantee that all TGFβ1 was removed from the hydrogel, fresh medium was added, removed after 2–3 hours and replaced with new fresh medium, which was changed every other day.

### Viability, proliferation and metabolic activity in 3D

Cell viability was evaluated using the Live/Dead assay. Cell-laden matrices were washed three times with DMEM/F-12 without phenol red (Gibco), then incubated (45 min, 37 °C in the dark) with calcein AM (1 μM, live cells) and ethidium homodimer-1 (EthD-1, 2.5 μM, dead cells) and washed again. Samples were imaged by confocal laser scanning microscopy (CLSM, Leica SP2 AOBS SE).

Cell proliferation was assessed by Ki-67 (Abcam, 1:100) immunostaining. EpH4-laden hydrogels were fixed with 4 wt.% paraformaldehyde (PFA, Sigma) in TBS-Ca (TBS with 7.5 mM CaCl_2_) for 20 min, permeabilized for 5 min with 0.2% v/v Triton X-100/TBS-Ca, and then incubated for 1 h in 5 wt.% bovine serum albumin (BSA) in TBS-Ca to block unspecific binding. Finally, samples were incubated with goat anti-rabbit secondary antibody Alexa Fluor 488 (Molecular Probes, Invitrogen, 1:1000, 1 h at RT) and nuclei were counterstained with DAPI.

Cell metabolic activity was assessed using the resazurin assay. Cell-laden matrices were incubated with 20% v/v of the stock resazurin solution (0.1 mg/mL, Sigma) in medium for 2 h at 37 °C. The supernatant was then transferred to a 96-well plate black with clear bottom (Greiner) and fluorescence measurements were carried out using a microplate reader (Biotek Synergy MX) with Ex/Em at 530/590 nm. For each condition n = 3 replicates were analyzed from 3 different biological replicas.

For total double-stranded DNA (dsDNA) quantification, the 3D matrices were dissolved and EpH4 cells were recovered by centrifugation (1500 rpm, 5 min) washed with PBS, centrifuged and stored at −20 °C until analyzed. Cells were lysed in 1% v/v Triton X-100 for 1 h at 250 rpm and 4 °C. Samples were then diluted 1:10 in PBS and used for dsDNA quantification using the Quant-iT PicoGreen dsDNA kit (Molecular Probes, Invitrogen), according to manufacturer’s instructions. Briefly, samples were transferred to a 96-well plate black with clear bottom and diluted in TE buffer (200mMTris–HCl, 20 mM EDTA, pH 7.5). After adding the Quant-iT PicoGreen dsDNA reagent, samples were incubated for 5 min at RT in the dark, and fluorescence was quantified using a microplate reader with Ex/Em at 480/520 nm. For each condition n = 3 replicates were analyzed from 3 different biological replicas.

### Analysis and quantification of spheroids formation

For analysis of spheroids formation at day 1, 7 and 14, EpH4 cells were pre-labeled with CellTracker Green CMFDA (Molecular Probes) at 15 μM for 30 min, and then entrapped within alginate matrices as described. Whole-mounted samples with fluorescence-labeled cells within alginate matrices were imaged by CLSM. The scanned Z-series were projected onto a single plane and colored using Fiji. For quantification of spheroids number and size during epithelial morphogenesis and EMT and its reversion, EpH4-laden hydrogels at different time points (E cells at day 1, 7 and 14) and at different states (E, M and RE) were fixed, permeabilized and blocked as described above. F-actin filaments were stained with Alexa Fluor 488 phalloidin (Invitrogen, 1:40 in 1 wt.% BSA/TBS-Ca, 1 h at RT) and nuclei were counterstained with DAPI. Samples were imaged by CLSM. The scanned Z-series were projected onto a single plane and spheroid diameter and number was assessed with Fiji. Three independent gels were analyzed for each condition and time point, and a total of 1876 and 1357 spheroids were analyzed for epithelial morphogenesis and EMT and its reversion, respectively.

### Histological analysis

For histology, EpH4-laden matrices (E state) at day 12 were dehydrated with a series of ethanol solutions of increasing concentrations and included in paraffin blocks. Thereafter, sections (3 μm) were recovered, de-paraffinized in Clear-Rite^TM^ 3 and rehydrated with a series of ethanol solutions of decreasing concentrations and finally water. For hematoxilin and eosin (H&E) staining, sections were incubated in Gill’s hematoxylin for 3 min and counterstained with Eosin Y for 2 min. Finally sections were dehydrated, de-paraffinized in Clear-Rite^TM^ 3 and mounted in glass slides using Entellan^®^ mounting medium. Samples were imaged by optical microscopy.

### Analysis of morphology, polarity, matrix deposition and phenotype

EpH4-laden matrices (E state) at day 12 were fixed, permeabilized and blocked as previously described. To analyze polarity, samples were incubated with the following primary antibodies overnight at 4 °C: rabbit anti-mouse ZO-1 (Invitrogen, 1:50) and goat anti-mouse β-catenin (BD Biosciences, 1:50). Afterwards, samples were incubated with goat anti-rabbit secondary antibody Alexa Fluor 488 and goat anti-mouse Alexa Fluor 594 (Molecular Probes, Invitrogen, 1:1000, 1 h at RT). To analyze the deposition of a laminin-rich layer, samples were incubated with primary rabbit anti-mouse laminin antibody (Sigma, 1:50, overnight at 4 °C) and then with goat anti-rabbit secondary antibody Alexa Fluor 488 (Molecular Probes, Invitrogen, 1:1000, 1 h at RT). In both cases, nuclei were counterstained with DAPI and samples were imaged by CLSM.

EpH4-laden matrices at different states (E, M and RE) were fixed, permeabilized and blocked as described in previous section. Subsequently, samples were incubated with the following primary antibodies: rabbit anti-mouse E-cadherin (Cell Signaling, 1:100, 2 hours at RT); rabbit anti-mouse fibronectin (Sigma, 1:100, ON at 4 °C) and rabbit anti-mouse vimentin (GenScript, 1:100, overnight at 4 °C). Finally, samples were incubated with goat anti-rabbit secondary antibody Alexa Fluor 594 (for E-cadherin) and 488 (for fibronectin and vimentin) (Molecular Probes, Invitrogen, 1:1000, 1 h at RT) and nuclei were counterstained with DAPI. Samples were imaged by CLSM and the scanned Z-series were projected onto a single plane and colored using Fiji. To assess lumen formation images were composed of only 1 Z-stack, which allows visualizing the central part of the spheroids, and thus lumens.

### RNA expression quantification

RNA was extracted from EpH4-laden matrices at the three different states (E, M and RE) (n = 4 biological replicas) using the Quick-RNA MiniPrep (Zymo Research), as recommended by the manufacturer. Subsequently, 1000 ng of total RNA were reversed transcribed to single stranded cDNA using Superscript First-strand synthesis synthesis and random hexamer primers (Invitrogen). Quantitative Real-Time PCR (qRT-PCR) was carried out using source RNA from 4 biological replicas for the target genes *CDH1*, *Ocln*, *CDH2*, *Zeb2*, *Mgat3*, *Id2* and for endogenous control GAPDH using as probe sets Mm00486909, Mm.PT.47.16166845, Mm00483212_m1, Mm.PT.47.13169136, Mm00447798_s1, Mm00711781_m1, 4352932 (Applied Biosystems and Integrated DNA Technologies), respectively. Samples were run in triplicates in the ABI Prism 7000 Sequence Detection System under the following conditions: 95 °C for 20 sec, followed by 40 cycles at 95 °C for 3 sec and 60 °C for 30 sec. Data was analyzed by the comparative 2(−ΔΔCT) method^63^.

### Matrix metalloproteinases (MMPs) secretion by zymography

To detect expression of MMPs (MMP2 and MMP9) activity by 3D cultured cells at different states (E, M and RE), cell culture supernatants were analyzed by gelatin-zymography from 3 biological replicas. After 72 h, conditioned media were collected, centrifuged to remove cell debris and loaded into gelatin-SDS polyacrylamide gels. Sample volumes were adjusted to yield equivalent total cell protein contents, which were quantified by the BCA Protein Assay (Pierce) in lysates of cells recovered from the hydrogels^62^. The gel was run in 1× Tris-Glycine SDS running buffer at 60 V (Mini Protean Tetra Cell system, BioRad). After electrophoresis, gels were washed twice and subsequently incubated in MMP substrate buffer for 16 h at 37 °C. Afterwards, gels were washed and stained with Coomassie Brilliant Blue R-250 (Sigma). MMPs proteolytic activity was visualized as the presence of clear bands against a blue background of Coomassie Blue-stained gelatin substrate. Thereafter, the gel was washed with distilled water, scanned using the GS-800 Calibrated Densiometer (Bio-Rad) and MMPs activity was estimated by densitometric analysis (Quantity One, Bio-rad).

### Evaluation of invasive potential of E, M and RE cells

E, M and RE cells were retrieved from alginate matrices upon dissolution with 0.05% w/v trypsin/50 mM EDTA solution, followed by centrifugation and incubation with collagenase II (1.5 mg/mL) for 30 min to dissociate cell spheroids. After centrifugation, cells were mixed with Matrigel Reduced Growth Factor (Corning) and 20 μL drops were pipetted into each well of a 24 well suspension culture plate (each EpH4-laden alginate gave rise to one EpH4-laden matrigel). After polymerization (30 min at 37 °C), fresh DMEM/F-12 was added to each sample. E and RE-laden matrigel were maintained in standard culture medium, while for M cells medium was supplemented with 16 ng/mL of TGFβ1. After 7 days, cell morphology was visualized under an inverted microscope (Axiovert 200 M, Zeiss). Metabolic activity was measured using the resazurin assay, immediately after entrapment and at day 7. For each condition n = 3 replicates were analyzed.

### Statistical analyses

Statistical analyses were performed using GraphPad Prism 6.0 software version 6.0 f. The non-parametric Mann–Whitney test was used to compare two groups. To compare spheroid diameter and number, Unpaired t test was used. Results for all analysis with ‘p’ value less than 0.05 were considered to indicate statistically significant differences (*p ≤ 0.05; **p < 0.01; ***p < 0.001).

## Additional Information

**How to cite this article**: Bidarra, S. J. *et al.* A 3D *in vitro* model to explore the inter-conversion between epithelial and mesenchymal states during EMT and its reversion. *Sci. Rep.* **6**, 27072; doi: 10.1038/srep27072 (2016).

## Supplementary Material

Supplementary Information
