# Supplementary material for: A 3D in vitro model to explore the inter-conversion between epithelial and mesenchymal states during EMT and its reversion
Source: Sci Rep. 2016 Jun 3;6:27072. doi: 10.1038/srep27072 (PMC4891772; doi:10.1038/srep27072)
Supplement: Supplementary Information [file srep27072-s1.pdf]

A 3D in vitro model to explore the inter-conversion between epithelial and mesenchymal states during EMT and its reversion

Bidarra SJ[1,2], Oliveira P[1,3,#], Rocha S[1,3,#], Saraiva DP[1,2], Oliveira C[1,3,4,+], Barrias CC[1,2,5,*,+]

1 - i3S – Instituto de Investigação e Inovação em Saúde, Universidade do Porto, Rua Alfredo Allen, 208, 4200-135 Porto, Portugal

2 - INEB - Instituto de Engenharia Biomédica

3 – Expression Regulation in Cancer Group, Institute of Molecular Pathology and Immunology of the University of Porto (IPATIMUP)

4 - Department of Pathology and Oncology, Faculty of Medicine, University of Porto, Al. Prof. Hernâni Monteiro, 4200-319 Porto, Portugal

5 – Instituto de Ciências Biomédicas Abel Salazar, Universidade do Porto, Rua de Jorge Viterbo Ferreira, 228, 4050-313 Porto, Portugal

* ccbarras@ineb.up.pt

[#]these authors contributed equally to this work

+these authors contributed equally as senior authors to this work

Supplementary Methods

Classification of the type of multicellular structures formed

To evaluate the type of multicellular structures formed by EpH4 in 3D after 12 days, paraffin-embedded sections stained with hematoxylin and eosin were used. Sections were prepared as previously described and imaged by optical microscopy. Images of three independent experiments were used to classify the structures formed by EpH4 cells in 3D into five different categories, as proposed by Weiss *et al.*: (I) immature with few cells, (II) spherical with a filled lumen, (III) spherical with a hollow lumen, (IV) non-spherical but organized, or (V) non-spherical and disorganized [29]. For each experiment a total of 500 structures were manually counted.

Analysis of spheroids formation

To investigate the mechanism of spheroids formation, EpH4 cells were divided in 3 different groups that were labeled with different CellTracker (Molecular Probes) dyes: Green CMFDA, Red CMTPX and Blue CMAC at 15 μM for 30 min. After centrifugation the 3 groups were mixed and labeled cells were entrapped within alginate hydrogel as described in section 2.3. Whole-mounted samples with fluorescence-labeled cells within alginate matrices were imaged by CLSM at day 1 and 14. The scanned Z-series were projected onto a single plane and colored using Fiji.

Supplementary Figures

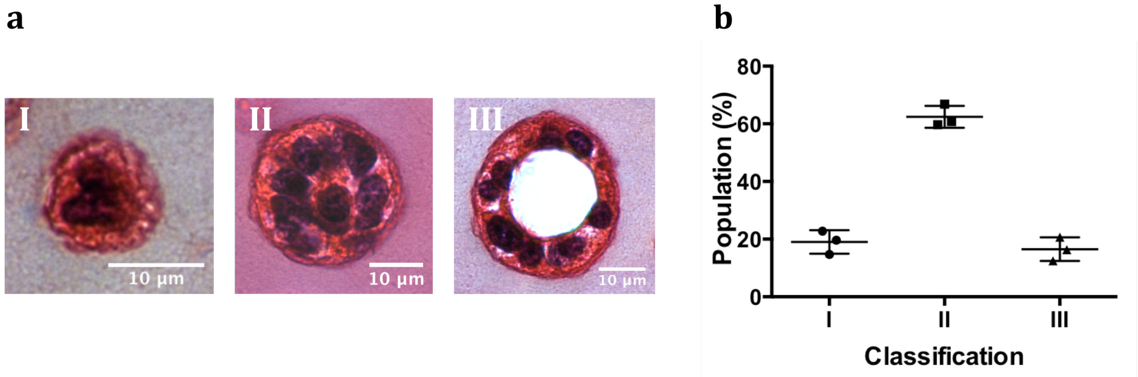

Figure S1. Classification of the type of multicellular structures formed by EpH4 within 3D alginate matrices. a) After 12 days of culture, 3 types of structures could be observed: (I) immature with few cells, (II) spherical with a filled lumen or (III) spherical with a hollow lumen. Scale bars: 10 μm. b) The percentage of each type of structure was quantified (3 independent experiments. In each, at least 500 structures were counted).

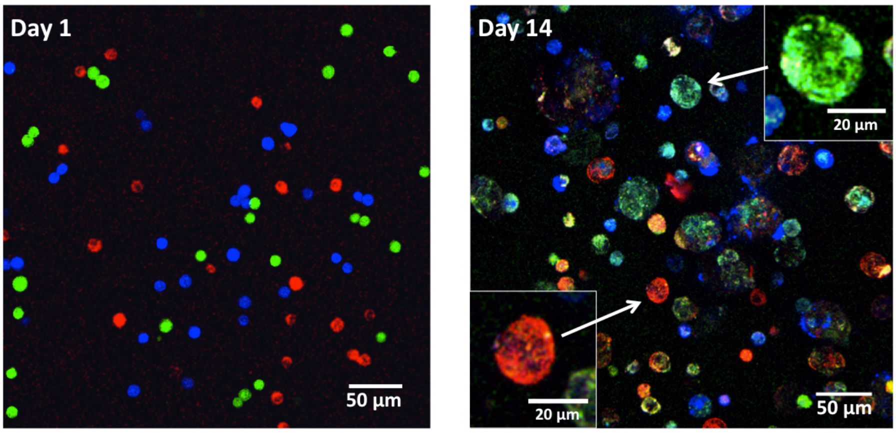

Figure. S2. Eph4 cells were labeled with CellTracker™ green, red and blue. The 3 different pre-labeled EpH4 cell populations were immobilized in 1%wt-RGD-alginate matrix as previously described. a) At day 1 individual pre-labeled cells were uniformly distributed within the matrix. b) After 2 weeks of culture, EpH4 cells formed single-coloured spheroids resulting from clonal-growth (inset images). Scale bars: 50 μm (inset images: 20 μm).
